# Predictors of the Healthy Eating Index and Glycemic Index in Multi-Ethnic Colorectal Cancer Families

**DOI:** 10.3390/nu10060674

**Published:** 2018-05-26

**Authors:** S. Pamela K. Shiao, James Grayson, Amanda Lie, Chong Ho Yu

**Affiliations:** 1 College of Nursing and Medical College of Georgia, Augusta University, Augusta, GA 30912, USA; 2 Hull College of Business, Augusta University, Augusta, GA 30912, USA; jgrayson@augusta.edu; 3 Citrus Valley Health Partners, Foothill Presbyterian Hospital, Glendora, CA 91741, USA; amandalie413@gmail.com; 4 School of Business, University of Phoenix, Pasadena, CA 91101, USA; alexyu@email.phoenix.edu

**Keywords:** healthy eating, glycemic index, colorectal cancer, generalized regression elastic net, diverse ethnic groups

## Abstract

For personalized nutrition in preparation for precision healthcare, we examined the predictors of healthy eating, using the healthy eating index (HEI) and glycemic index (GI), in family-based multi-ethnic colorectal cancer (CRC) families. A total of 106 participants, 53 CRC cases and 53 family members from multi-ethnic families participated in the study. Machine learning validation procedures, including the ensemble method and generalized regression prediction, Elastic Net with Akaike’s Information Criterion with correction and Leave-One-Out cross validation methods, were applied to validate the results for enhanced prediction and reproducibility. Models were compared based on HEI scales for the scores of 77 versus 80 as the status of healthy eating, predicted from individual dietary parameters and health outcomes. Gender and CRC status were interactive as additional predictors of HEI based on the HEI score of 77. Predictors of HEI 80 as the criterion score of a good diet included five significant dietary parameters (with intake amount): whole fruit (1 cup), milk or milk alternative such as soy drinks (6 oz), whole grain (1 oz), saturated fat (15 g), and oil and nuts (1 oz). Compared to the GI models, HEI models presented more accurate and fitted models. Milk or a milk alternative such as soy drink (6 oz) is the common significant parameter across HEI and GI predictive models. These results point to the importance of healthy eating, with the appropriate amount of healthy foods, as modifiable factors for cancer prevention.

## 1. Introduction

Colorectal cancer (CRC) is recognized as the most preventable cancer worldwide [1]. Unhealthy dietary habits with excess caloric intake and weight gain, smoking, and over-consuming alcohol can increase the risk of developing CRC [2,3,4,5,6] through inflammatory oxidative stress pathways [7,8,9,10,11,12]. Furthermore, based on the strong evidence defined by The American Institute for Cancer Research (AICR), plant-based foods and healthy weight, reducing red meat and alcohol intake [1,13] can prevent CRC. Fortunately, dietary habits can be improved as one of the modifiable lifestyle factors to prevent CRC and cancer progression [6,7,8,9,10,11,12].

A healthy diet has been associated with decreased CRC risk, examined by using the healthy eating index (HEI) [14,15,16,17,18]. Elements of a healthy diet include adequate intakes of vegetables and dark green vegetables, fruits and whole fruits, grains and whole grains, nuts and legumes, proteins including fish and other seafood, milk or alternative dairy products for lactose intolerance; and limiting salt, saturated fat, and empty calories from sugar and alcohol [2,3,4,5,7,8]. Higher HEI scores are associated with decreased CRC risk [19,20,21]. Additionally, diets rich in fiber, folate, calcium, limiting pro-inflammatory fatty acids are protective against CRC [22,23,24]. The glycemic index (GI) has been used to assess healthy eating in association with CRC, to manage hyperinsulinemia and insulin resistance [25]. A low-GI with low glycemic load (GL) diet may decrease inflammation and CRC risk [26,27,28,29,30]. For prevention, the risk of CRC was reduced by half when participants followed 4–6 recommendations of healthy eating components over 8 years [31]. 

In summary, dietary habits are formed over time within families, and the family units can share both dietary habits as part of lifestyle [22,23,24,32] and the heredity of genome and epigenetics of CRC [33,34,35]. Family-based studies can provide potential insights into developing prevention strategies for cancer prevention. Therefore, the aim of this study, following a previous report on gene-environment interactions in a family-based study involving CRC patients and their family members [32], was to investigate and predict healthy eating practices by HEI and GI from various dietary and demographic factors of the multi-ethnic CRC families. In this study, we used machine learning validation procedures including the ensemble method [36,37,38,39] and generalized regression prediction, Elastic Net with Akaike’s Information Criterion with correction and Leave-One-Out cross validation methods [40,41,42,43]. 

## 2. Materials and Methods

### 2.1. Study Population and Setting

The study methods were reported before [32] and are summarized in the following. We included 106 participants, 53 CRC cases and 53 family members by accessing the California Cancer Registry (CCR) database and other cases through referrals from the community that the study was conducted. The designated Human Subjects Institutional Review Boards (IRB) from the local educational institutions and the California State Committee for the Protection of Human Subjects approved the project [32]. With the approved study procedures, the qualified participants were recruited. The participants were interviewed on campus or in their homes. 

### 2.2. Demographic Data

Demographic data included lifestyle and dietary status [32,44], family history, functional capacities using the items included in the 1999–2012 National Health Interview Survey [45] and the family pedigrees from the Coalition for Health Professional Education in Genetics ([46], www.nchpeg.org).

### 2.3. Dietary Indexes

We assessed healthy eating by using dietary measurements including HEI (HEI-2015) [16,18], GI [47,48] and recommended daily intakes (RDI) [17], collected with Food Frequency Questionnaire [49,50] and data processed through the Nutrition Data Systems for Research [51,52]. HEI was developed to assess diet quality issued by the US Department of agriculture (USDA) based on the standards of a healthy lifestyle in association with health outcomes. HEI is composed of 12 scored components which include 5 major food groups: fruit (total and whole), vegetable (total and greens/beans), grains (total and whole), dairy or alternative dairy and protein, oils and nuts; in addition to limiting saturated fats, sodium, and empty calories. The total HEI score is the sum of the components, with a range of 0 to 100. A score between 0–50 indicates a poor diet; 51–80, a moderate diet quality that needs improvement; and a score greater than 80, a good diet [16,53].

GI is a measure of carbohydrates in foods on a scale of 0–100, based on how the foods affect the levels of blood sugar. Foods with a high GI (score of 70 or more) are quickly digested, absorbed and metabolized, causing a quick spike in blood sugar and insulin levels. A low GI diet (score of 55 or less) includes whole grains or carbohydrates that lead to a slow and steady release of blood sugar and insulin [54]. Examples of foods with high GI include white bread, pretzels, potatoes, corn flakes, and foods with lower GI include whole wheat bread, rolled or steel-cut oatmeal, sweet potatos, legumes, non-starchy vegetables. One study systematically organized GI values for over 1000 foods [55]. GL takes into consideration the GI in foods (http://lpi.oregonstate.edu/mic/food-beverages/glycemic-index-glycemic-load). GL is calculated by multiplying the GI by the quantity (grams) of carbohydrates in a serving of a food divided by 100 (≤10: low, 11–19: medium, ≥20: high [56].

The recommended daily intake (RDI) is issued by the Food and Nutrition Board of the Institute of Medicine, which recommends the sufficient required daily intake of nutrients for healthy people based on gender and age [17]. Macronutrients include carbohydrates, protein, total fat, saturated fat, cholesterol; B vitamins—B9 (folate), B1 (thiamine), B2 (riboflavin), B3 (niacin) B6 and B12; and other micronutrients—Vitamin A, C, D and E, calcium, magnesium, iron, zinc, methionine, and choline [57] 

### 2.4. Data Analysis

Machine learning based analytics were employed in JMP Pro 13 ([58,59,60], SAS Institute, Cary, NC, USA). The analytics and rationales have been reported earlier [32] and are summarized in the following. We included ensemble methods [36,37,38,39], for a well-known remedy in small-sample studies [61] with random subsets of repeated analysis to correct bias [62], which is superior to conventional regression modeling for a best fit model [63,64]. We used generalized regression (GR) with machine learning validation to obtain a smaller prediction error [43]. It is important to point out that GR eliminates certain predictors to avoid over-fitting. For example, when there are several collinear predictors, LASSO selects only one and ignore the others or zeroes out some regression coefficients. The Ridge method counteracts against collinearity and variance inflation by shrinking the regression coefficients towards zero, but not exactly zero. The Elastic Net method combines the penalties of the LASSO and Ridge approaches. Unlike linear least squares in estimating the unknown parameters in a linear regression model, GR could simply zero out certain unused predictors [60]. In traditional statistics, usually one model is used to fit the data, and thus the probability is nothing more than an approximation based on sampling distributions, which are open-ended (the two-tails never touch the x-axis). In this case, the *p* value at most could only be 0.9999, but not exactly one. However, when all permutations are exhausted, such as what was done in an exact test, the probability could be exactly one. In a similar vein, GR exhausts different paths to find the best model. When the full model has a mixture of important and unused predictors, the *p* value cannot be one. However, when the data could be perfectly described by the restricted model resulting from path searching, the probability of observing the data could be 1. 

When developing a GR model for a predictive model, the first type of model presented in JMP Pro 13 is a logistic regression (LR) model because the default estimation method is an LR. After this default method, other model launches can be pursued by choosing a variety of estimation methods (lasso, Elastic Net and others) and associated validation methods (a validation column, minimum AICc, leave-one-out (LOO) validation and others, [65]). Both AICc validation and LOO cross-validation methods are effective methods for small data sets [66]. In effect, the default LR method could be characterized as an explanatory model whereas the other GR estimation methods might best be characterized as a predictive model. An explanatory model is typically used to explain the association between the model parameters and the model response to test causal hypotheses, using a predictive model, for predicting future observations [67]. The nature of the model objectives (causal versus predictive) directly influence the underlying algorithms which can result in different results of models using the same set of initial parameters. Typically, using an explanatory model, a set of statistically significant parameters is identified for a final model. The predictive model using GR will pursue methods to shrink coefficients towards zero in part to guard against overfitting the model. For model prediction in GR analysis, continuous variables are recoded into new dichotomous variables grouped by either median distribution or a known score criterion of healthy eating. The prediction profiler and interactive profiler can be used to visualize the direction of association between two parameters (a predictor or factor with the outcome variable of healthy eating status in profiler) or among three parameters (set of interactive variables with non-parallel distribution in addition to the outcome status of healthy eating in the interactive profiler). The visualization of the profiler and interactive profiler will enable the analyst to visualize and account for the interactions of various factors. The index of showing the fitness of the model over complexity is AIC or AICc [64,65,66,67,68,69,70], with a smaller AIC suggesting a more optimal model for model quality [68,71,72]. We examined model quality using the misclassification rate (smaller is better), AICc, and the area under the receiver operating characteristic (ROC) curve (AUC). 

## 3. Results

### 3.1. Characteristics of Study Participants

Table 1 presents the key demographic characteristics of the 106 participants. There were more women than men in the sample, with racial compositions of about one-third Asians, one-third Caucasians, and one-third Hispanic and African Americans combined. About 25% of the sample presented as obese based on body mass index (BMI), more than half of the sample drank alcohol, and 8.5% were smokers. 

### 3.2. Dietary Parameters

The distribution on the dietary parameters was organized and presented for HEI and GI parameters in Table 2. Overall, this sample presented a healthy eating profile based on the average recommended intake for the HEI parameters; however, the average of limiting parameters (saturated fat, salt, and empty calorie) was higher than recommended levels, with less than half of the sample (38.7%) receiving a good HEI score of greater than 80. The median HEI score for this sample was 77, with 51% of the sample above the median score. The average GI was 53.8, which presents as a low GI diet (good GI), with 62% of the sample scoring less than 55 (Table 2). 

RDI parameters are presented in Table 3. For carbohydrates, 71% of the sample consumed more than 45% of the RDI. For protein, 36% of the sample ate more than 20% of the RDI. For saturated fat, 48% of the sample consumed less than 10% of RDI. On average, the sample consumed more than the RDI for all parameters except cholesterol, fiber, total folate, calcium and magnesium. For cholesterol, the mean intake was 259 mg, and 74% of the sample ate less than 100% of the RDI (<300 mg). For fiber, the mean intake was 19 g; 15% of the sample ate more than 100% of the RDI (≥25 g). In addition, only 32% of the sample had more than 100% of the RDI for total folate (>400 μg), with an average mean intake of 365.5 μg. Less than half of the sample consumed more than 75% of the RDI for calcium (1000 mg) and magnesium (320 mg), with mean intakes of 837 mg and 295 mg, respectively. For sodium, the mean intake was 2950 mg, which was greater than the RDI of <2300 mg, and only 38% of the sample ate less than 100% of the RDI (Table 3). 

### 3.3. Predictive Modeling for Healthy Eating—Generalized Regression Analysis

Four sets of models were tested for prediction of healthy eating based on HEI and GI scores: an HEI score greater than 80 (HEI 80) is a good HEI score, an HEI score of 77 and higher (HEI 77) is the median score for this sample, GI of 55 and lower (low and good GI), and GI of 53.8 (median score for this sample). All individual dietary parameters under HEI and RDI categories and demographic parameters were tested for variables of importance and predictive models. Eleven common parameters across the four scoring criteria (HEI 80, HEI 77, GI 55, and GI 53.8) were identified for the prediction of healthy eating. These 11 parameters include in sequence of presentation in these analyses: whole fruit (1 cup), milk or dairy alternative such as a soy drink (6 oz), whole grain (1 oz), saturated fat (15 g), oils and nuts (1 oz), empty calories (300), fiber (19 g), gender, gender interacting with cancer status (Group Ca), Group Ca, and dark greens (6 oz) (Supplementary Tables S1–S4). We presented the testing on all 11 common parameters in addition to the models with significant parameters to illustrate the differences between the models with misclassification rates for accuracy of prediction, AICc for fitness, and AUC for coverage.

**Figure 1:**
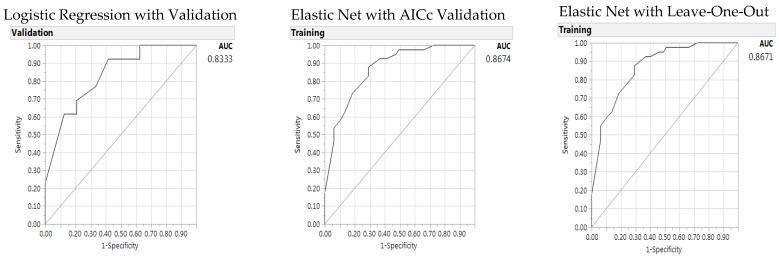
Predictors of the Healthy Eating Index (80): Area under the receiver operating characteristic curve (AUC) for logistic regression (**left**), Elastic Net with Akaike’s information criteria with correction (AICc) validation (**middle**) and Leave-One-Out validation models (**right**).

Table 4 presents significant individual parameters for HEI 80 prediction. A baseline LR model with validation was constructed with five significant individual parameters; all five parameters are HEI items: whole fruit (1 cup), milk or soy drink (6 oz), whole grain (1 oz), saturated fat (15 g), and oil nut intakes (1 oz) (all *p* < 0.05, amount per component representing the medians of this sample), with no significant parameters from other categories of demographic or RDI parameters. The results of baseline LR with validation are shown in the left panel of Table 4. Then, two GR models were developed using Adaptive Elastic Net with AICc validation and LOO cross validation methods to predict the probability of healthy eating with HEI 80 (the middle and right panels of Table 4). In both GR validation models, oil and nut intake did not present statistical significance. The GR AICc validation model presented as the best model with lowest misclassification rate and highest AUC, but higher AICc than the baseline LR model. The AUC as shown in Figure 1 with the baseline LR model presented 0.8333 and the GR Elastic Net AICc model and LOO model with AUC of 0.8674 and 0.8671, respectively. 

Compared to the 11-parameter model that included all significant parameters for all models combined (Supplementary Table S1, Supplementary Figure S1), the 5-parameter model in Table 4 presented better model quality with smaller AICc (better) with fewer parameters (58 versus 75 for LR and 105 versus 113 for the GR AICc validation) and lower misclassification rate (better) (0.30 versus 0.32 for LR). The 5- and 11-parameter models presented similar AUC across the LR and GR models, with increased (better) AUC for the LR model. 

**Figure 2:**
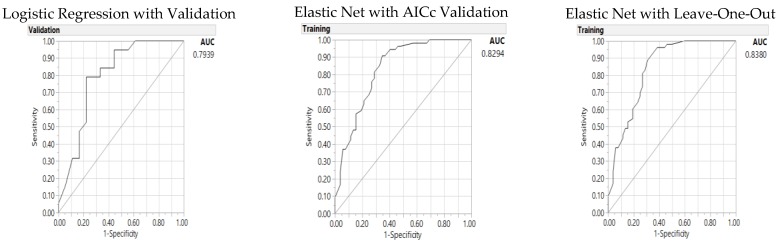
Predictors of the Healthy Eating Index (77): Area under the receiver operating characteristic curve (AUC) for baseline logistic regression (**left**), Elastic Net with Akaike’s information criteria with correction (AICc) validation (**middle**) and Leave-One-Out validation models (**right**).

These models are then tested with the HEI score of 77 (HEI 77) as the median score of HEI for this study sample (Table 5). There is one significant interaction in addition to the six individual parameters in the model for HEI 77 (Table 5): milk or soy drink (6 oz), whole grain (1 oz), empty calories (300), and fiber (19 g) as dietary parameters; gender and cancer/control status, and interaction of gender and cancer/control status. While cancer/control status as an individual parameter is not significant with respect to the *p* value, it must be included in the model because of its significant interaction with the gender status. The GR LOO validation model presents as the best model with the highest number of significant parameters, lowest misclassification rate for accuracy and highest AUC (Figure 2). 

In comparison to the 11-parameter model (Supplementary Table S2 and Figure S2), the significance model in Table 5 presents better fitness with lower AICc (63 versus 69 for LR); while the 11-parameter models present lower misclassification rates for both GR models (0.1604 versus 0.25 for the GR AICc validation and 0.1524 versus 0.23 for the GR LOO validation) and higher AUCs (0.86 versus 0.79 for LR, 0.90 versus 0.83 for GR AICc, and 0.92 versus 0.84 for GR LOO models). In comparison to the HEI 80, HEI 77 presented with lower misclassification rates, but higher AICc and lower AUC across LR and GR models. 

**Figure 3:**
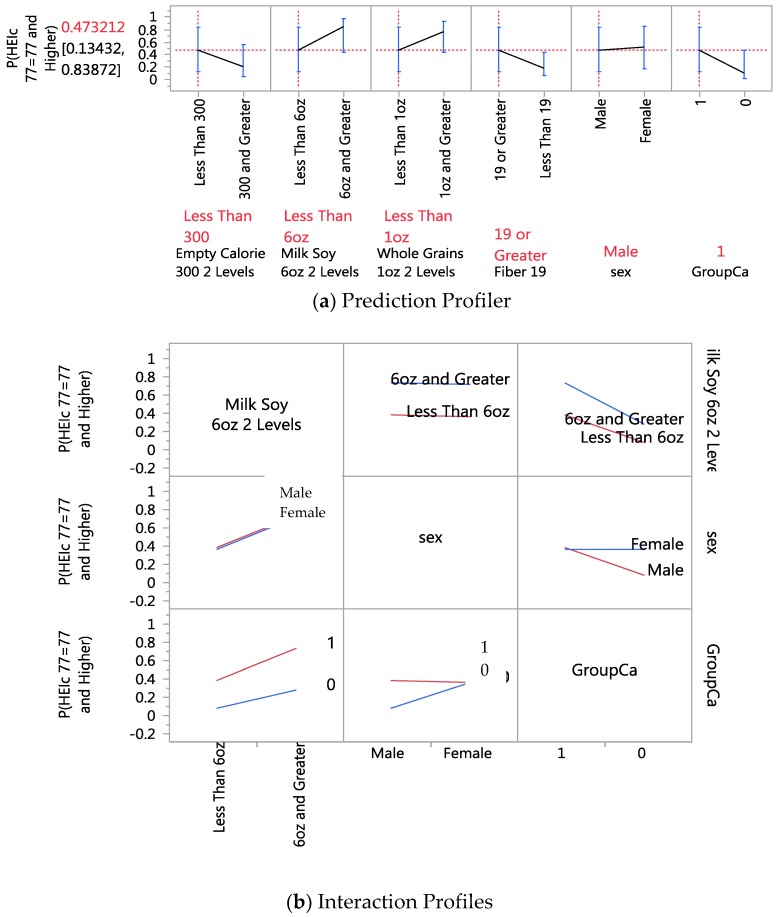
Prediction profiler (**a**) for significant predictors of health eating (score 77) and (**b**) interaction of gender with cancer/control group (non-parallel and crossing lines) when compared to another parameter (dairy or soy drink intake) without interaction (parallel lines).

The JMP profiler, shown in Figure 3a, and the interaction profiler shown in Figure 3b, are illustrative of how to interpret the interaction results. To illustrate, the excerpt of the interaction profiler depicts interactions between milk soy and gender, gender and cancer/control group status (group Ca), milk soy and cancer/control group status. Visually, the more non-parallel the two levels, the more likely there is a significant interaction between the two parameters. For example, we see in the milk soy and gender cell the lines or levels are almost parallel, indicating likely no-significant interaction. However, for the gender with group Ca, there is a crossing of the two lines, indicating there is likely a statistically significant interaction effect between these parameters; a significant finding in the GR LOO validation (*p* < 0.05).

**Figure 4:**
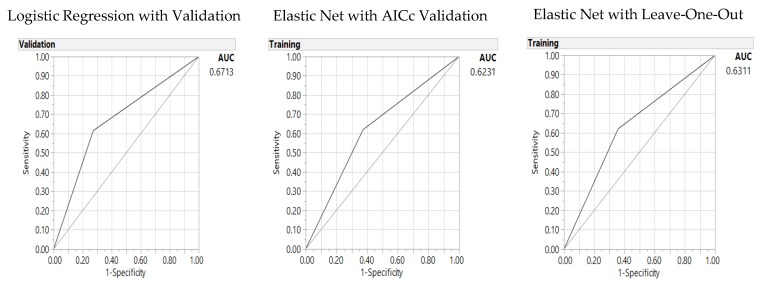
Predictors of the Glycemic Index (55): Area under the receiver operating characteristic curve (AUC) for baseline logistic regression (**left**), Elastic Net with Akaike’s information criteria with correction (AICc) validation (**middle**) and Leave-One-Out validation models (**right**).

The models are then tested with the GI score of 55 (GI 55), as the good GI score (Table 6). There is only one significant parameter: milk or soy drink in this model. LR outperformed two GR validation models for this one significant parameter model with the lowest misclassification rate, lower AICc, and highest AUC (Figure 4). 

In comparison to the 11-parameter model (Supplementary Table S3 and Figure S3), the significance model in Table 6 presents better fitness with lower AICc (49 versus 87 for LR, and 139 versus 153 for GR AICc validation); while the 11-parameter models present lower misclassification rates for both GR models (0.16 versus 0.25 for the GR AICc validation and 0.15 versus 0.23 for the GR LOO validation) and higher AUCs (0.86 versus 0.79 for LR, 0.90 versus 0.83 for GR AICc, and 0.92 versus 0.84 for GR LOO models) and AUC for LR (0.67 versus 0.55). 

**Figure 5:**
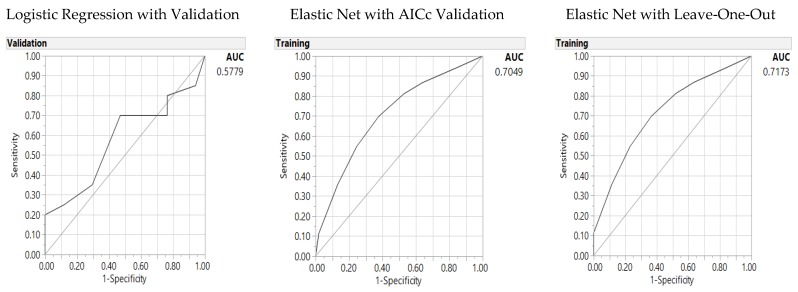
Predictors of the Glycemic Index (53.8): Area under the receiver operating characteristic curve (AUC) for baseline logistic regression (**left**), Elastic Net with Akaike’s information criteria with correction (AICc) validation (**middle**) and Leave-One-Out validation models (**right).**

Finally the models are then tested with the GI score of 53.8 (GI 53.8), as the median GI score (Table 7). Three dietary parameters under the HEI domain categories were significant parameters for GI 53.8: milk or soy drink empty calories, and dark greens. GR validation outperformed the LR model with lower misclassification rates and higher AUC (Figure 5).

In comparison to the 11-parameter model (Supplementary Table S4 and Figure S5), the significance model in Table 7 presents better fitness with lower AICc with fewer parameters in the model (62 versus 91 for LR, and 141 versus 149 for GR AICc validation) and slightly higher AUC for GR LOO model (0.717 versus 0.715; while the 11-parameter models present slightly higher AUC (0.63 versus 0.58 for LR, 0.72 versus 0.70 for GR AICc). In comparison to the GI 55 prediction, GI 53.8 predictive models present lower misclassification rates across LR and GR models and higher AUC for GR models. However, GI 55 models present lower AICc in both LR and GR AICc validation models with fewer parameters. In comparison with the two HEI models of HEI 80 and HEI 77, two GI models of GI 55 and GI 53.8 presented higher misclassification rates, higher AICc, and lower AUC across all LR and GR models; hence, the HEI models presented better quality models than the GI models.

## 4. Discussion

We presented a ground-breaking study, to cross-validate the results using both conventional LR statistics, with machine learning-based analytics, including the ensemble method and GR validation methods to predict healthy eating in diverse multi-ethnic families with CRC patients. While previous studies presented higher HEI scores in association with lower risks of CRC [19,20,21,73], we further documented the sensitivity of the HEI scale with median split distribution (a score of 77 versus 80) for predictive testing of healthy eating in association with CRC risk. Predictors of HEI 80 as the criterion score of a good diet included five significant dietary parameters (with intake amount): whole fruit (1 cup), milk or alternative-soy drinks for lactose intolerance (6 oz) [74], whole grain (1 oz), saturated fat (15 g), and oil and nuts (1 oz) for the diverse multi-ethnic sample of CRC families. Compared to the GI models, HEI models presented more accurate, fitted models, and greater coverage. Milk or alternative dairy for lactose intolerance [74] such as soy drinks (6 oz) is the common significant parameter across four HEI and GI predictive models. 

Using SAS JMP programming (SAS Institute, Cary, NC, USA), we identified significant parameters of healthy eating in the diverse groups of families of CRC patients with their family members. As dietary habits can be modified, specific domain parameters for healthy eating can be helpful for these families to focus on key food items, with specific amounts for minimum intake levels or restricted intake levels. For a demonstration study of future dietary interventions, we used machine learning-based analytics, including ensemble methods and GR AICc and LOO validation models, for small-sample studies to validate the analyses by the random subsets of samples [75]. We further presented an interaction profiler including 3-way interactions (interaction profile includes bi-variate interactions in association with the outcome) for the best quality and optimal model. 

As part of prevention efforts, healthy eating is essential in personalized nutrition for nutrigenetics in providing methyl-donors to prevent CRC. Family members share dietary habits and lifestyles that affect epigenetics and nutrigenomics pathways affecting health outcomes [76]. For sustainable improvement of dietary modifications, as part of healthy lifestyles, the involvement of family members is vital to provide an essential support system within the families with heightened awareness of healthy eating within the family units [32,76]. Further studies with larger datasets and diverse samples are needed to further examine these findings in diverse groups for personalized nutrition in preparation for precision-based healthcare.

## Figures and Tables

**Table 1:** Demographic characteristics of the sample.

Parameters		Total (*N* = 106) *n* (%)
Gender	Male	39 (37%)
	Female	67 (63%)
Age, Years	M ± SD	54 ± 16
Ethnicity	Asian	40 (38%)
	Caucasian	34 (32%)
	Hispanic	23 (22%)
	African American	9 (9%)
BMI status	Obese	26 (25%)
Alcohol drinker	Yes	57 (54%)
Smoker	Yes	9 (9%)

Note: BMI: body mass index.

**Table 2:** Healthy Eating Index and parameters for the sample (*N* = 106).

Parameters (Amount, Maximum Score)	Intake M ± SD	Score M ± SD	Maximum Score *n* (%)
Calorie (per day)	1600 ± 850	--	--
Total Fruit (≥0.8 cup, 5 points)	1.6 ± 1.5	4.1 ± 1.5	69 (65%)
Whole Fruit (≥0.4 cup, 5 points)	1.2 ± 1.1	4.2 ± 1.5	78 (74%)
Vegetables (≥1.1 cup, 5 points)	1.5 ± 1.2	4.1 ± 1.4	62 (59%)
Dark greens (≥0.4 cup, 5 points)	0.9 ± 0.7	4.4 ± 1.2	77 (73%)
Total Grain (≥3 oz, 5 points)	4.6 ± 3.0	4.3 ± 1.2	66 (62%)
Whole Grain (≥1.5 oz, 5 points)	1.7 ± 1.7	3.4 ± 1.7	45 (43%)
Dairy (≥1.3 cup, 10 points)	1.4 ± 3.2	5.4 ± 3.6	24 (23%)
Protein (≥2.5 oz, 10 points)	5.8 ± 4.2	9.2 ± 2.0	86 (54%)
Oil and nuts (≥12 g, 10 points)	36 ± 22	9.9 ± 0.4	103 (97%)
Saturated Fat (g, ≤8% energy)	18.5 ± 11.4	7.0 ± 2.7	18 (17%)
Sodium (≤1.1 g, 10 points)	3.2 ± 1.9	2.1 ± 3.5	6 (6%)
Empty Calorie (≤19% energy, 20 points)	350 ± 230	17.6 ± 3.4	47 (44%)
Healthy Eating Index score (>80, good)	76 ± 9	--	>80: 41 (39%)
≥77 (median distribution)			≥77: 54 (51%)
Glycemic Load	96 ± 59	--	--
Glycemic Index (≤55, low and good)	54 ± 4.2	--	≤55: 66 (62%)
≤53.8 (median distribution)			≤53.8: 53 (50%)

Note: M: mean, SD: standard deviation, oz: ounce, g: gram.

**Table 3:** Recommended dietary daily intake for the sample (*N* = 106).

Parameters, Unit, RDI	M ± SD	Intake %	*n* (%)
Carbohydrates, g, 45–65% calorie	200 ± 110	≥45%	75 (71%)
Protein, g, 10–35% calorie	77 ± 44	≥20%	38 (36%)
Total Fat, g, 20–35% calorie	370 ± 220	<35%	69 (65%)
Saturated Fat, g, <10% calorie	19 ± 11	<10%	51 (48%)
Cholesterol, <300 mg	260 ± 170	<100%	78 (74%)
Sodium, <2300 mg	3000 ± 1700	<100%	40 (38%)
Fiber, ≥25 g	19 ± 10	≥100%	16 (15%)
Total Folate, 400 μg	370 ± 220	≥100%	34 (32%)
Vitamin B1 (Thiamine), 1.1 mg	1.4 ± 0.8	≥100%	65 (61%)
Vitamin B2 (Riboflavin), 1.1 mg	1.9 ± 1.3	≥100%	78 (74%)
Vitamin B6, 1.3 mg	1.8 ± 1.0	≥100%	68 (64%)
Vitamin B12, 2.4 μg	6.1 ± 8.2	<150%	44 (42%)
Niacin, 14 mg	21 ± 12	≥100%	72 (68%)
Calcium, 1000 mg	840 ± 620	≥75%	46 (43%)
Magnesium, 320 mg	300 ± 160	≥75%	52 (49%)
Iron, 8 mg	13 ± 7.6	≥100%	44 (42%)
Zinc, 8 mg	11 ± 6.9	≥100%	53 (50%)
Methionine, 13 mg/Kg	1.8 ± 1.0	<150%	45 (43%)

Note: RDI: recommended daily intake, g: gram; mg: milligram, μg: microgram, Kg: Kilogram.

**Table 4:** Predictors of Healthy Eating Index (80): Baseline logistic regression and generalized regression Elastic Net models.

	Logistic Regression with Validation		Generalized Regression Elastic Net			
			AICc Validation		Leave-One-Out Validation	
Parameters	Estimate	*p* (*X*^2^)	Estimate	*p* (*X*^2^)	Estimate	*p* (*X*^2^)
(Intercept)	2.64	0.002	1.61	0.001	1.58	0.002
Whole Fruit 1 cup	−2.51	0.002	−1.90	0.0004	−1.86	0.0006
Milk Soy 6 oz	−2.62	0.002	−1.86	0.0002	−1.84	0.0002
Whole Grain 1 oz	−2.44	0.007	−2.28	0.001	−2.26	0.002
Sat Fat 15 g	3.81	0.008	2.31	0.010	2.55	0.010
Oil Nut 1 oz	−2.57	0.02	−1.29	0.12	−1.56	0.09
Misclassification Rate	0.30		0.23		0.23	
AICc	58		105		n/a	
Area under the curve	0.83		0.87		0.87	

Note. AICc: Akaike’s information criterion with corrections.

**Table 5:** Predictors of Healthy Eating Index (77): Baseline logistic regression and generalized regression Elastic Net models.

	Logistic Regression with Validation		Generalized Regression Elastic Net			
			AICc Validation		Leave-One-Out Validation	
Parameters	Estimate	*p* (*X*^2^)	Estimate	*p* (*X*^2^)	Estimate	*p* (*X*^2^)
(Intercept)	1.11	0.18	0.27	0.65	0.31	0.61
Milk Soy 6 oz	−2.23	0.0008	−1.82	0.0003	−1.71	0.0006
Whole Grain 1 oz	−1.23	0.10	−1.30	0.02	−1.37	0.01
Empty Calories 300	1.21	0.11	1.21	0.03	1.10	0.048
Fiber 19 g	1.01	0.21	1.36	0.03	1.38	0.03
Gender	−2.04	0.11	−1.83	0.06	−2.60	0.003
GroupCa * Gender	1.88	0.23	1.63	0.17	2.73	0.03
GroupCa	−0.54	0.47	0.37	0.55	0.29	0.63
Misclassification Rate	0.27		0.25		0.23	
AICc	63		122		n/a	
Area under the curve	0.80		0.83		0.84	

Note. AICc: Akaike’s information criterion with corrections * interaction.

**Table 6:** Predictors of the Glycemic Index (55): Baseline logistic regression and generalized regression Elastic Net models.

	Logistic Regression l with Validation		Generalized Regression Elastic Net			
			AICc Validation		Leave-One-Out Validation	
Parameters	Estimate	*p* (X^2^)	Estimate	*p* (X^2^)	Estimate	*p* (X^2^)
(Intercept)	0.73	0.04	1.07	0.0005	1.01	0.0009
Milk Soy 6 oz	−0.86	0.09	−1.07	0.01	−1.01	0.02
Misclassification Rate	0.35		0.37		0.38	
AICc	49		139		n/a	
Area Under Curve	0.67		0.62		0.63	

Note. AICc: Akaike’s information criterion with corrections.

**Table 7:** Predictors of Glycemic Index (53.8): Baseline logistic regression and generalized regression Elastic Net models.

	Logistic Regression with Validation		Generalized Regression Elastic Net			
			AICc Validation		Leave-One-Out Validation	
Parameters	Estimate	*p* (X^2^)	Estimate	*p* (X^2^)	Estimate	*p* (X^2^)
(Intercept)	0.51	0.34	0.79	0.04	0.87	0.03
Milk Soy 6 oz	−1.36	0.02	−1.29	0.003	−1.41	0.002
Empty Calories 300	1.37	0.02	0.62	0.16	0.74	0.10
Dark Green 6 oz	−1.18	0.04	−0.94	0.03	−1.05	0.02
Misclassification Rate	0.38		0.34		0.33	
AICc	62		141		n/a	
Area Under Curve	0.58		0.70		0.72	

Note. AICc: Akaike’s information criterion with corrections.

## Supplementary Materials

The following are available online at http://www.mdpi.com/2072-6643/10/6/674/s1, Table S1: Table 1. Predictors of Healthy Eating Index (80): Baseline logistic regression and generalized regression Elastic Net models including 11 common parameters, Table S2: Predictors of Healthy Eating Index (77): Baseline logistic regression and generalized regression Elastic Net models including 11 common parameters, Table S3: Predictors of Glycemic Index (55): Baseline logistic regression and generalized regression Elastic Net models including 11 common parameters, Table S4: Predictors of Glycemic Index (53.8): Baseline logistic regression and generalized regression Elastic Net models including 11 common parameters, Figure S1: Predictors of Healthy Eating Index (80), including 11 common parameters: Area under the receiver operating characteristic curve (AUC) for baseline logistic regression model (left panel), Elastic Net with Akaike’s information criteria with correction (AICc) validation model (middle) and Leave-One-Out validation model (right panel), Figure S2: Predictors of Healthy Eating Index (77), including 11 common parameters: Area under the receiver operating characteristic curve (AUC) for baseline logistic regression model (left panel), Elastic Net with Akaike’s information criteria with correction (AICc) validation model (middle) and Leave-One-Out validation model (right panel), Figure S3: Predictors of Glycemic Index (55) including 11 common parameters: Area under the receiver operating characteristic curve (AUC) for baseline logistic regression model (left panel), Elastic Net with Akaike’s information criteria with correction (AICc) validation model (middle) and Leave-One-Out validation model (right panel), Figure S4: Predictors of Glycemic Index (53.8) including 11 common parameters: Area under the receiver operating characteristic curve (AUC) for baseline logistic regression model (left panel), Elastic Net with Akaike’s information criteria with correction (AICc) validation model (middle) and Leave-One-Out validation model (right panel).
