# Supplementary material for: Predictors of the Healthy Eating Index and Glycemic Index in Multi-Ethnic Colorectal Cancer Families
Source: Nutrients. 2018 May 26;10(6):674. doi: 10.3390/nu10060674 (PMC6024360; doi:10.3390/nu10060674)
Supplement: Supplementary file 1 [file nutrients-10-00674-s001.pdf]

Supplementary File: Predictors of Healthy Eating Index and Glycemic Index in Multi-Ethnic Colorectal Cancer Families

S. Pamela K. Shiao*, James Grayson, Amanda Lie, Chong Ho Yu; *Corresponding: pshiao@msn.com

Supplementary Table 1. Predictors of Healthy Eating Index (80): Baseline logistic regression and generalized regression Elastic Net models including 11 common parameters.

	Logistic Regression Model with Validation		Generalized Regression Elastic Net Model			
			AICc Validation		Leave-One-Out Validation	
Parameters	Estimate	*p* (X^2)	Estimate	*p* (X^2)	Estimate	*p* (X^2)
Whole Fruit 1 cup	-2.03	0.01	-1.62	0.003	-1.57	0.002
Milk Soy 6oz	-2.41	0.003	-1.79	0.001	-1.62	0.001
Whole Grains 1oz	-1.59	0.08	-1.85	0.01	-1.80	0.01
Sat Fat 15g	3.45	0.03	1.96	0.05	1.52	0.10
Oil Nut 1oz	-2.46	0.07	-1.26	0.16	-0.71	0.42
Fiber 19	1.85	0.01	1.11	0.01	1.12	0.01
Empty Calorie 300	1.61	0.14	1.02	0.20	0.69	0.36
Gender	-0.99	0.54	-0.76	0.36	0	1.00
GroupCa*Gender	1.23	0.52	0.92	0.27	0.05	0.93
GroupCa	-0.08	0.93	0	1.00	0	1.00
Dark Green 6oz	0.64	0.42	0.73	0.19	0.71	0.15
Misclassification	0.32		0.23		0.21	
AICc	75		113		n/a	
Area Under Curve	0.82		0.87		0.88	

Note. AICc: Akaike's information criterion with corrections.

Supplementary Table 2. Predictors of Healthy Eating Index (77): Baseline logistic regression and generalized regression Elastic Net models including 11 common parameters.

	Logistic Regression Model with Validation		Generalized Regression Elastic Net Model			
			AICc Validation		Leave-One-Out Validation	
Parameters	Estimate	*p* (X^2)	Estimate	*p* (X^2)	Estimate	*p* (X^2)
Whole Fruit 1 cup	-2.07	0.01	-2.05	0.001	-2.28	0.001
Milk Soy 6oz	-2.19	0.01	-1.71	0.002	-1.83	0.001
Whole Grains 1oz	-1.48	0.08	-1.63	0.01	-1.82	0.002
Sat Fat 15g	1.57	0.25	0.83	0.46	0.74	0.37
Oil Nut 1oz	0.10	0.93	0.06	0.96	0	1.00
Fiber 19	2.07	0.01	1.74	0.004	1.91	0.001
Empty Calorie 300	1.70	0.10	1.45	0.06	1.48	0.06
Gender	-2.03	0.22	-1.44	0.07	-0.99	0.17
GroupCa*Gender	1.93	0.32	1.19	0.31	0.28	0.80
GroupCa	0.07	0.93	0.62	0.48	1.38	0.09
Dark Green 6oz	0.88	0.27	0.86	0.15	1.18	0.03
Misclassification	0.27		0.16		0.15	
AICc	69		113		n/a	
Area Under Curve	0.86		0.90		0.92	

Note. AICc: Akaike's information criterion with corrections.

Supplementary Table 3. Predictors of Glycemic Index (55): Baseline logistic regression and generalized regression Elastic Net models including 11 common parameters.

	Logistic Regression Model with Validation		Generalized Regression Elastic Net Model			
			AICc Validation		Leave-One-Out Validation	
Parameters	Estimate	p (X^2)	Estimate	p (X^2)	Estimate	p (X^2)
Whole Fruit 1 cup	0.61	0.31	0.76	0.09	0.48	0.22
Milk Soy 6oz	-1.39	0.03	-1.12	0.02	-0.96	0.02
Whole Grains 1oz	-0.07	0.91	0.20	0.73	0	1.00
Sat Fat 15g	0.48	0.63	-0.64	0.43	0	1.00
Oil Nut 1oz	0.13	0.88	0.98	0.23	0.57	0.29
Fiber 19	1.33	0.03	1.00	0.03	0.86	0.03
Empty Calorie 300	1.05	0.17	0.73	0.17	0.44	0.36
Gender	-1.09	0.27	-1.35	0.07	-0.74	0.23
GroupCa*Gender	0.69	0.60	0.94	0.33	0.14	0.83
GroupCa	0.03	0.96	-0.09	0.88	0	1.00
Dark Green 6oz	-0.63	0.28	-0.19	0.68	0	1.00
Misclassification	0.35		0.33		0.30	
AICc	87		153		n/a	
Area Under Curve	0.55		0.69		0.70	

Note. AICc: Akaike's information criterion with corrections.

Supplementary Table 4. Predictors of Glycemic Index (53.8): Baseline logistic regression and generalized regression Elastic Net models including 11 common parameters.

	Logistic Regression Model with Validation		Generalized Regression Elastic Net Model			
			AICc Validation		Leave-One-Out Validation	
Parameters	Estimate	p (X^2)	Estimate	p (X^2)	Estimate	p (X^2)
Whole Fruit 1 cup	0.30	0.64	0.22	0.63	0	1.00
Milk Soy 6oz	-1.45	0.02	-1.42	0.001	-1.25	0.002
Whole Grains 1oz	0.63	0.38	0.25	0.63	0	1.00
Sat Fat 15g	-0.69	0.52	0	1.00	0	1.00
Oil Nut 1oz	0.68	0.47	0.54	0.35	0.58	0.12
Fiber 19	1.04	0.23	0.26	0.66	0	1.00
Empty Calorie 300	1.75	0.04	0.26	0.62	0	1.00
Gender	-0.51	0.61	0	1.00	0	1.00
GroupCa*Gender	1.05	0.44	0.59	0.22	0.37	0.44
GroupCa	0.12	0.87	0	1.00	0	1.00
Dark Green 6oz	-1.22	0.06	-1.08	0.02	-0.97	0.02
Misclassification	0.35		0.36		0.34	
AICc	91		149		n/a	
Area Under Curve	0.63		0.72		0.72	

Note. AICc: Akaike's information criterion with corrections.

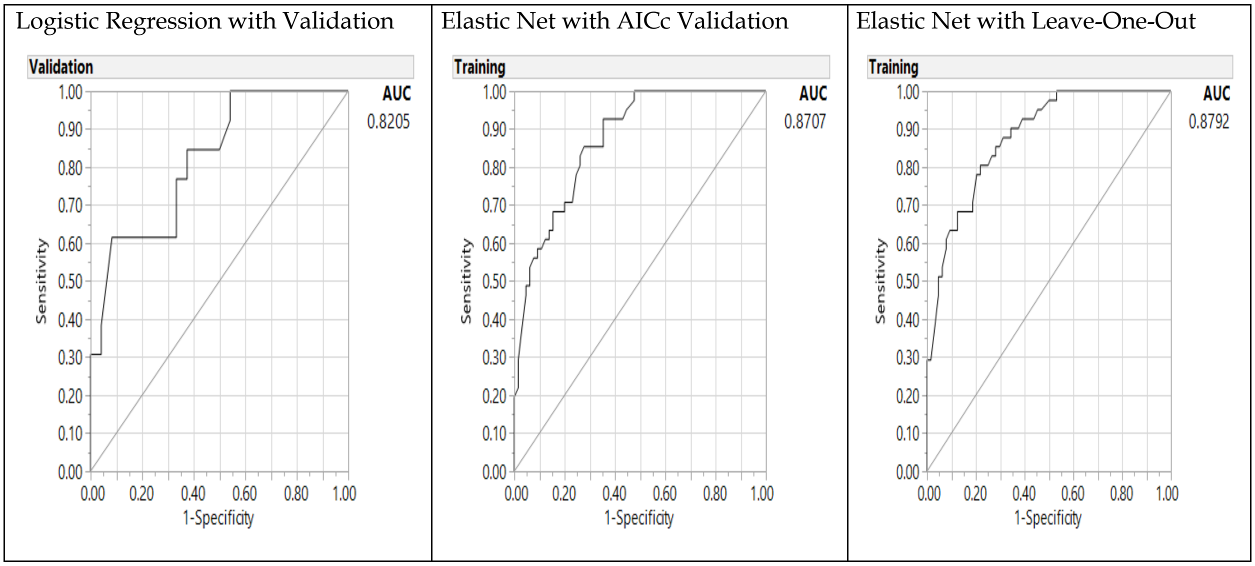

Supplementary Figure 1. Predictors of Healthy Eating Index (80), including 11 common parameters: Area under the receiver operating characteristic curve (AUC) for baseline logistic regression model (left panel), Elastic Net with Akaike's information criteria with correction (AICc) validation model (middle) and Leave-One-Out validation model (right panel).

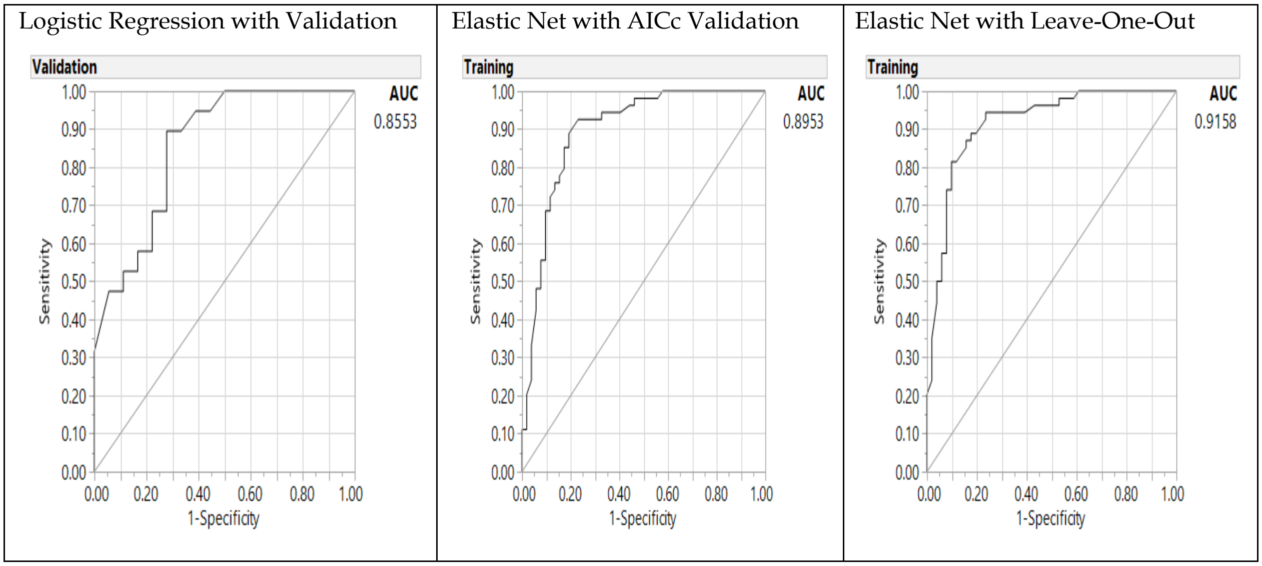

Supplementary Figure 2. Predictors of Healthy Eating Index (77), including 11 common parameters: Area under the receiver operating characteristic curve (AUC) for baseline logistic regression model (left panel), Elastic Net with Akaike's information criteria with correction (AICc) validation model (middle) and Leave-One-Out validation model (right panel).

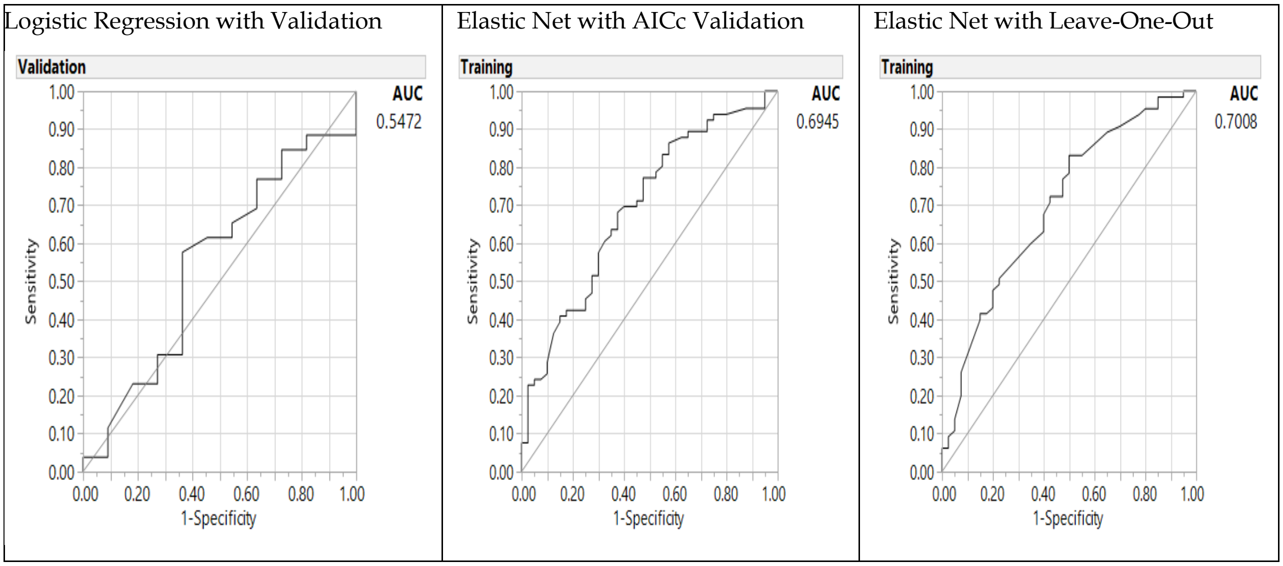

Supplementary Figure 3. Predictors of Glycemic Index (55) including 11 common parameters: Area under the receiver operating characteristic curve (AUC) for baseline logistic regression model (left panel), Elastic Net with Akaike's information criteria with correction (AICc) validation model (middle) and Leave-One-Out validation model (right panel).

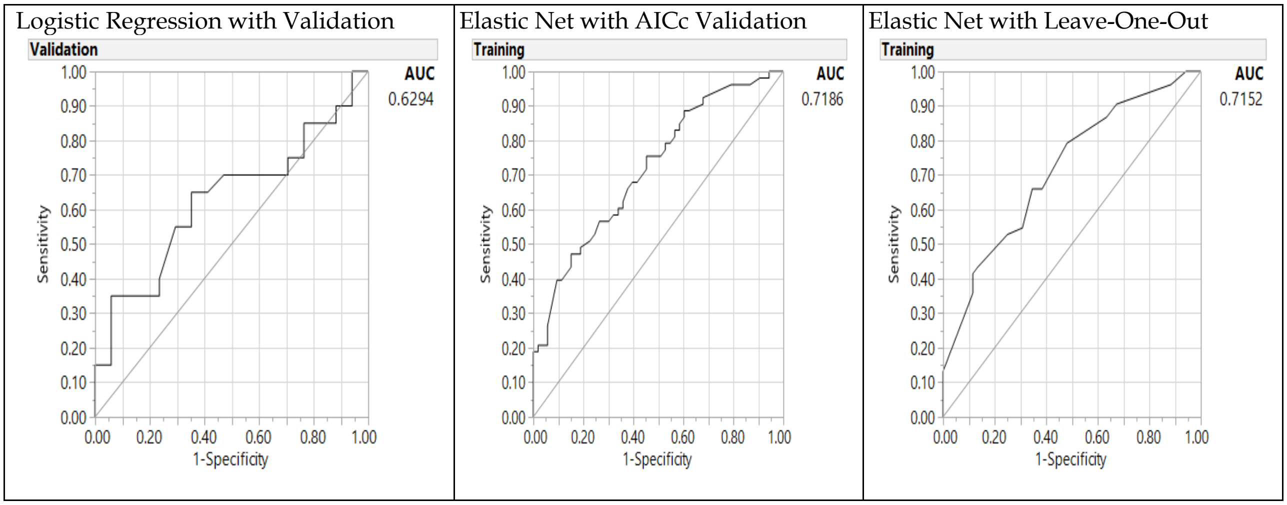

Supplementary Figure 4. Predictors of Glycemic Index (53.8) including 11 common parameters: Area under the receiver operating characteristic curve (AUC) for baseline logistic regression model (left panel), Elastic Net with Akaike's information criteria with correction (AICc) validation model (middle) and Leave-One-Out validation model (right panel).
